# Basic life support, a necessary inclusion in the medical curriculum: a cross-sectional survey of knowledge and attitude in Uganda

**DOI:** 10.1186/s12909-022-03206-z

**Published:** 2022-03-03

**Authors:** Nelson Ssewante, Godfrey Wekha, Angelique Iradukunda, Phillip Musoke, Andrew Marvin Kanyike, Germinah Nabukeera, Nicholas Kisaakye Wamala, Wilson Zziwa, Lauben Kamuhangire, Jonathan Kajjimu, Tonny Stone Luggya, Andrew Tagg

**Affiliations:** 1 School of Medicine, College of Health Sciences, Makerere University, Kampala, Uganda; 2 Faculty of Health Sciences, Busitema University, Mbale, Uganda; 3 School of Medicine, Kabale University, Kabale, Uganda; 4 Faculty of Clinical Medicine and Dentistry, Kampala International University, Ishaka-Bushenyi, Uganda; 5 Faculty of Medicine, Gulu University, Gulu, Uganda; 6 Faculty of Biology, Medicine, and Health, King Caesar University, Kampala, Uganda; 7 Faculty of Medicine, Mbarara University of Science and Technology, Mbarara, Uganda; 8 Department of Anesthesia and Emergency Medicine, Makerere University, Kampala, Uganda; 9 Emergency Department, Western Hospital-Footscray, Footscray, Victoria Australia; 10 School of Medicine, University of Melbourne, Melbourne, Victoria Australia

**Keywords:** Knowledge, Attitude, Basic life support, Medical student, Emergency response, Medical curriculum

## Abstract

**Background:**

Uganda continues to depend on a health system without a well-defined emergency response system. This is in the face of the rising cases of out-of-hospital cardiac arrest contributed largely to the high incidence of road traffic accidents. Non-communicable diseases are also on the rise further increasing the incidence of cardiac arrest. Medical students are key players in the bid to strengthen the health system which warrants an assessment of their knowledge and attitude towards BLS inclusion in their study curriculum.

**Methods:**

A descriptive cross-sectional study was conducted in 2021 among undergraduate medical students across eight public and private universities in Uganda. An online-based questionnaire was developed using Google forms and distributed via identified WhatsApp groups. Chi-square or Fisher’s exact test and logistic regression were performed in STATA 15 to assess the association between knowledge of BLS and demographics. P < 0.05 was considered statistically significant.

**Results:**

Out of the total 354 entries obtained, 351 were analyzed after eligibility screening. Of these, (*n* = 250, 71.2%) were male less than 25 years (*n* = 273, 77.8%). Less than half (*n* = 150, 42.7%) participants had undergone formal BLS training.

Less than a third of participants (*n* = 103, 29.3%) had good knowledge (≥ 50%) with an overall score of 42.3 ± 12.4%. Age (*p* = 0.045), level of academic progress (*p* = 0.001), and prior BLS training (*p* = 0.033) were associated with good knowledge. Participants with prior training were more likely to have more BLS knowledge (aOR: 1.7, 95% CI: 1.1–2.7, *p* = 0.009).

The majority (*n* = 348, 99.1%) believed that BLS was necessary and would wish (*n* = 343, 97.7%) to have it included in their curriculum.

**Conclusions:**

Undergraduate medical students have poor BLS knowledge but understand its importance. Institutions need to adopt practical teaching methods such as clinical exposures, field experience in collaboration with local implementers, and participating in community health promotion campaigns.

**Supplementary Information:**

The online version contains supplementary material available at 10.1186/s12909-022-03206-z.

## Background

The provision of Basic Life Support (BLS) involves the recognition of sudden cardiac arrest followed by the activation of an emergency response system, early cardiopulmonary resuscitation (CPR), and rapid defibrillation with an automated external defibrillator [1]. This can be provided by a health professional or any other trained bystander. Cardiac arrest is an increasingly common life-threatening event that accounts for more than 7 million deaths globally per year [2]. Common causes of cardiac arrest include cardiovascular diseases such as ischemic heart disease, cardiomyopathy, and valvular heart diseases among others accelerated by sedentary lifestyles, smoking, alcohol, and physical inactivity [3]. Trauma is another cause of cardiac arrest that cannot be over-emphasized especially in resource-limited countries where emergency response services are not well established [4, 5].

Uganda loses more than 35,000 individuals to road traffic accidents each year, largely due to delays in the initiation of BLS and transportation of victims to health facilities [6, 7]. Additionally, the lack of confidence in the provision of BLS contributed in part by lack of formal training and refresher courses, even in-hospital CPR is often delayed [8]. Reports indicate that only 18.4% of patients who suffered from a cardiac arrest in Mulago hospital received CPR [9]. This communicates a gap in the training programs that inadequately prepare health workers in emergency response. The heavy patient load owed to the alarmingly low doctor-patient ratio in Uganda may be another important factor explaining this deficit in health performance [10]. In the setting where there’s a paucity of trained bystanders, measures to better health professionals’ performance in the emergency care of patients must be taken meticulously. Such measures must be aimed at health system strengthening on both short- and long-term basis an example being goal-oriented medical training. This approach not only answers the question of competent health workers but also contributes to system strengthening by the provision of sufficient human resource necessary for training willing bystanders [11]. Some Universities such as Makerere University incorporate some aspects of first aid-BLS training in particular course units but knowledge of the practicability and retention of the information obtained from such training is not well studied. Therefore, this study aimed to assess the knowledge and attitude towards BLS among undergraduate medical students in Uganda to inform on the feasibility of the current medical schools’ curricula and how these can be improved to suit the country’s health care needs.

## Methods

### Study design and area

This was a cross-sectional quantitative study that targeted undergraduate medical students across eight public and private universities in Uganda from April to May 2021. The public universities include; Makerere University, Mbarara University of Science and Technology, Gulu University, Busitema University, and Kabale University. The private universities involved were; Kampala International University, Islamic University in Uganda, and King Caesar International University.

### Study population

Undergraduate medical students undertaking Bachelor of Medicine and Bachelor of Surgery (MBChB), and other related courses including Bachelor of Nursing, Bachelor of Dental Surgery, Bachelor of Pharmacy at any of the aforementioned universities.

### Selection criteria

All undergraduate medical students aged 18 years and above, enrolled in one of the above universities and undertaking the above-mentioned courses were eligible to participate in this survey. Being purely an online study, respondents without internet access were automatically excluded.

### Sample size calculation

The sample size was calculated using Epi Info StatCalc for infinite population surveys. A 5% acceptable margin of error was considered; design effect of 1.0; cluster effect of 1.0, and a power of 80%, giving us an estimated sample size of 384 participants at a 95% confidence interval (95% CI). To cater for non-response associated with online surveys, 10% of the estimated sample size was added leading to a final sample size of 422 participants.

### Sampling procedure and data collection

Uganda was in a partial lockdown at the time this study was conducted, with schools and higher institutions of learning using a blended physical and e-learning system to maximize social distancing. Because of this, we opted to use social media platforms such as WhatsApp Messenger® for enrolling eligible participants through contact persons identified at the different participating universities using a convenience sampling technique.

A questionnaire was developed using Part 3 of the 2015 International Consensus on Cardiopulmonary Resuscitation and Emergency Cardiovascular Care Science guidelines [12]. A link to the Google form was sent to the potential participants via the identified WhatsApp groups and data collated. Sensitive details such as names, email addresses, registration numbers were not collected to ensure anonymity.

### Data analysis

Data cleaning, coding, and marking were done using Microsoft Excel 2016 and exported to STATA® 15 for analysis. Demographic characteristics, knowledge, and attitude of the participants towards BLS were first summarized as frequencies and percentages for categorical variables, and means for numerical variables. Knowledge score was calculated by awarding a point for every correct response to questions with a maximum score of 18 points. They were then converted to percentages. Knowledge was graded into good (≥ 50%) or poor knowledge (< 50%). Chi-square or Fisher’s exact test and binary logistic regression were performed to assess the association between knowledge on BLS and demographics. P < 0.05 was considered statistically significant.

## Results

### Characteristics of participants

**Fig. 1:**
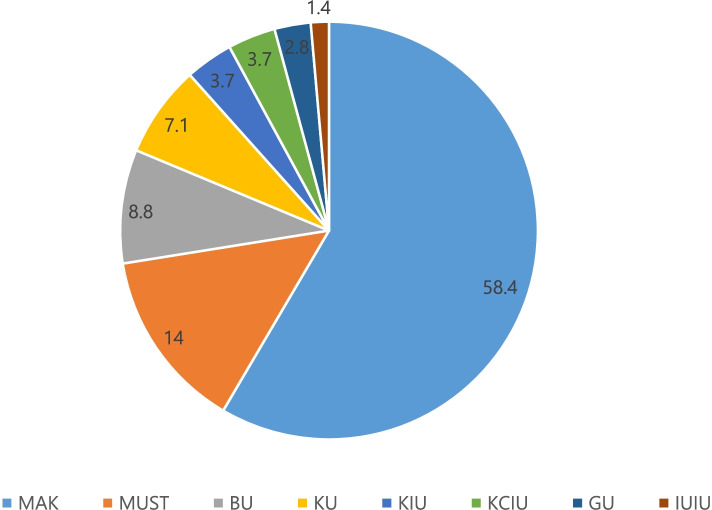
Distribution of participants across the different universities in Uganda. MAK: Makerere University, MUST: Mbarara University of Science and Technology, BU: Busitema University, KU: Kabale University, KCIU: King Caesar International University, KIU: Kampala International University, GU: Gulu University and IUIU: Islamic University in Uganda

A total of 354 entries were obtained out of which three were from ineligible courses and were excluded. Finally, 351 fully completed entries were cleaned and analyzed. Of the analyzed, (*n* = 250, 71.2%) were males and aged 25 years or less (*n* = 273, 77.8%). Most participants were undertaking MBChB (*n* = 301, 85.8%) mostly at a public university (*n* = 320, 91.2%). The distribution of participants in the different eligible universities in Uganda is shown in Fig. 1 Most of the participants were in clinical years of their training (*n* = 246, 70.1%). Only 150(42.7%) of the participants had undertaken BLS training before the study. Table 1 summarizes the baseline characteristics of participants.

**Table 1:** Characteristics of participants

Variable	Frequency	Percentage
**Age**		
≤ 25 years	273	77.8
> 25 years	78	22.2
**Sex**		
Female	101	28.8
Male	250	71.2
**University ownership**		
Public University	320	91.2
Private University	31	8.8
**Study program**		
MBChB	301	85.8
Others	50	14.3
**Level of academic progress**		
Preclinical stage	105	29.9
Clinical stage	246	70.1
**Prior BLS training**		
No	201	57.3
Yes	150	42.7

## Knowledge of basic life support

Of the 351 participants, less than a third (*n* = 103, 29.3%) had good knowledge regarding BLS (a score of > 50%). The mean knowledge score of all participants was 42.3% (SD = 12.4%). Participants above 25 years (46 vs. 41.2), MBChB students (42.9 vs. 38.4), Clinical students (43.6 vs. 39.2), and those with prior BLS training (44.7 vs. 40.4) had more mean scores compared to their counterparts.

At bivariate analysis, age (*p* = 0.045), level of academic progress (*p* = 0.001), and prior BLS training (*p* = 0.033) were significantly associated with good BLS knowledge. There was no association of university ownership (*p* = 0.650), sex (*p* = 0.541), or study program (*p* = 0.065) with BLS knowledge as shown in Table 2.

**Table 2:** Participants’ knowledge of BLS

Variable	BLS knowledge			
	Good, n (%)	Poor, n (%)	*p*-value	Mean score (SD)
Overall	103(29.3)	248(70.7)		42.3(12.4)
**Age**			0.045	
>25 years	30(38.5)	48(61.5)		46.0(12.0)
≤25 years	73(26.7)	200(73.3)		41.2(12.4)
**Sex**			0.541	
Female	32(31.7)	69(68.3)		42.5(12.6)
Male	71(28.4)	179(71.6)		42.2(12.4)
**University ownership**			0.650	
Private Universities	8(25.8)	23(74.2)		41.8(10.2)
Public Universities	95(29.7)	225(70.3)		42.3(12.6)
**Study program**			0.065	
MBChB	94(31.2)	207(68.8)		42.9(12.4)
Others	9(18.0)	41(82.0)		38.4(12.1)
**Level of academic progress**			0.001	
Clinical stage	85(34.6)	161(65.4)		43.6(12.5)
Preclinical stage	18(17.1)	87(82.9)		39.2(11.7)
**Prior BLS training**			0.033	
No	50(24.9)	151(75.1)		40.4(11.9)
Yes	53(35.3)	97(64.7)		44.7(12.7)

After adjusting for sex, university ownership, and level of academic progress, participants who had prior BLS training were more likely to be knowledgeable about BLS compared to those who had never received any formal training (OR: 1.7, 95% CI: 1.1–2.7, *p* = 0,018) as shown in Table 3.

**Table 3:** Multivariate logistic regression analysis of factors associated with BLS knowledge

Variables	Adjusted Odds Ratio (aOR)	95% CI	*p*-value
**Age**			
≤ 25 years	Reference		
> 25 years	1.6	0.9–2.6	0.082
**Sex**			
Male	Reference		
Female	1.1	0.7–1.8	0.577
**University Ownership**			
Public	Reference		
Private	1.3	0.6–2.8	0.497
**Study program**			
MBChB	Reference		
Others	0.5	0.3-1.0	0.051
**Level of academic progress**			
Pre-clinical stage	Reference		
Clinical stage	1.3	0.8–2.1	0.299
**Prior BLS training**			
No	Reference		
Yes	1.7	1.1–2.7	0.018

### Attitude towards Basic Life Support

**Fig. 2:**
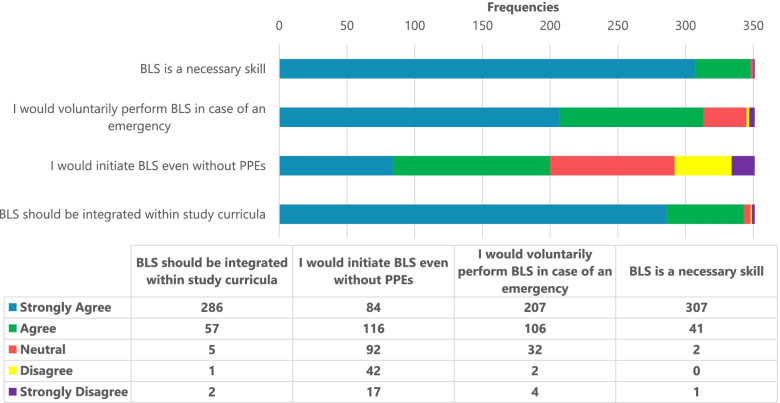
Attitude towards Basic Life Support among undergraduate medical students in Uganda

Overall, participants self-reported a good attitude towards BLS (Fig. 2). The majority of participants, either strongly agreed (*n* = 307, 87.5%) or agreed (*n* = 41, 11.7%) that BLS knowledge was a necessary skill; (*n* = 313, 89.2%) felt like they would voluntarily perform BLS on any victim who needed it while (*n* = 343, 97.7%) would wish to see BLS included within their study curriculum.

## Discussion

In the current study, we found that the majority of students lacked knowledge regarding the provision of BLS despite recognizing its importance. Prior BLS training was a significant predictor of good knowledge.

Like in many other parts of the world, our study revealed poor BLS knowledge with a mean knowledge score of 42.3 ± 12.4%. In a related study conducted in Saudi Arabia among medical students, the overall mean knowledge score of 32.7% was reported [13]. However, in Ethiopia, Tsegaye et al., reported that the majority of their participants had good knowledge about CPR [14]. The probable explanation for the difference in results of their study and ours is that whereas we recruited students in the preclinical and clinical years, participants in the Ethiopian study were all in clinical years. Therefore, it is possible that they got training in basic life support as they advanced through medical training or rather participated in emergency response during their clinical rotations. This was also evidenced in our study by the differences in the mean knowledge scores between clinical and pre-clinical years (41.1 vs. 46.1). The proportion of participants with formal BLS training was very low in our study as it has been reported in many other countries [8, 14–17]. This training not only improves the BLS knowledge but is also associated with better practices among beneficiaries. This is especially important in Uganda’s health system where general practitioners (including intern doctors and medical officers) are the main primary caregivers let alone the limited number of critical care specialists [18]. This has been adopted by the developed countries as a way of reducing mortality associated with OHCA through upscaling the number of trained bystanders at any available opportunity [19–21]. In a study assessing CPR knowledge and attitude among secondary school students in Norway, it was reported that up to 335(89%) of participants had been trained to perform CPR, mostly from schools and a few by organizations like the Red Cross [22]. This gives their health system good backup as it is estimated that providing bystander CPR to a victim of cardiac arrest can reduce the rate of deterioration from 10% to just 3% per minute [23].

Although the current medical curriculum may contain some aspects of BLS, the role of this component need review to ensure the taught skills are understood and can be practiced when needed. BLS is a practical subject whose components cannot be fully exhausted in a classroom. This is because, although using this approach may provide knowledge to the students, without the real-time hands-on practice of the acquired skills, this knowledge is prone to be forgotten. Literature shows that 50% of skills learned after BLS training are lost in the subsequent two months [24]. This is especially true where regular practice and undertaking refresher courses are not possible [25–29]. In a study assessing CPR knowledge and skills among health professionals in Tanzania [8], it was reported that knowledge scores declined with working experience, and those who regularly performed CPR had better knowledge scores. Additionally, our study also shows that participants undertaking MBChB had more knowledge scores compared to other courses in the current study. This may be due to the false belief that doctors are responsible for handling emergencies that need BLS skills which creates an unnecessary gap in the health system’s capacity to provide on-site BLS services.

Many training approaches have been assessed to determine the most efficient but the most common challenge for all has been difficulties in knowledge and skills retention [30–32]. Among the studied, self-directed learning methods and traditional teaching methods have been reported to be effective at skilling trainees if well supported by refresher training to improve knowledge and skills retention [33]. These can be incorporated into the current students’ training curriculum to impart the desired knowledge and skills to trainees. Intending to upscale the trained bystanders and the quality of emergency response in the country, medical students by virtue of their training and exposure are more suitable candidates to set a pace to achieve this milestone. Fortunately, the current study as do many previous studies in other countries, show that medical students have an excellent attitude towards BLS and are more than willing to take on BLS training. Since they are more likely to have opportunities for practicing the acquired skill sets, an important aspect in knowledge retention, the frequency of required refresher training would be lower increasing the feasibility of this approach.

To the best of our knowledge, this is the first study to assess the knowledge and attitude of BLS among undergraduate medical students in Uganda. Findings from this study suggest the need to strengthen the current curriculum to accommodate more practical ways of teaching BLS among medical students. Information from this study, though focused on medical students, may also be used to revise curricula in other faculties as a way of increasing the prevalence of trained bystanders in the country.

### Limitations

Being an online study conducted in the middle of the COVID-19 pandemic, data collection was only possible via online methods. Therefore, potential participants who did not have supporting gadgets, poor connectivity to the internet, or those who could not afford data costs for participating in this study were excluded. This might have led to selection bias which may limit the generalization of our study findings. Additionally, since the questionnaire was self-administered, there was a possibility of obtaining correct answers without a full understanding of the questions, recall bias and participants may have interpreted the questions differently.

## Conclusions

We found that majority of undergraduate students in Uganda lack BLS knowledge and prior training was the most important factor significantly associated with better BLS knowledge. Regardless of this, participants had an excellent attitude towards BLS training and wished to have it integrated into their curriculum. Academic institutions under the stewardship of the National Council for Higher Education (NCHE) and Uganda Medical and Dental Practitioners’ Council (UMDPC) should aim to adopt more practical approaches to BLS training. These may include but are not limited to; (1) organizing clinical exposure sessions after theory sessions involving real-time patient management, (2) collaborating with local implementers such as the Red Cross and St. John’s Ambulance to give medical students the necessary hands-on experience in the field, and (3) engaging medical students in community health promotion campaigns where they are empowered to teach the general population basic CPR provision.

Finally, further studies should be conducted to determine the feasibility of integrating BLS in non-medical curricula. This would increase the number of trained providers available to initiate CPR in the community.

## Supplementary Information

**Additional file 1.**

## Data Availability

The datasets used and/or analyzed during the current study are available from the corresponding author on reasonable request.

## Abbreviations

aOR: Adjusted Odds Ratio
BLS: Basic Life Support
CPR: Cardiopulmonary Resuscitation
MBChB: Bachelor of Medicine and Bachelor of Surgery
NCHE: National Council for Higher Education
OHCA: Out-of-hospital cardiac arrest
UMDPC: Uganda Medical and Dental Practitioners’ Council
