# Supplementary material for: Basic life support, a necessary inclusion in the medical curriculum: a cross-sectional survey of knowledge and attitude in Uganda
Source: BMC Med Educ. 2022 Mar 3;22:140. doi: 10.1186/s12909-022-03206-z (PMC8892119; doi:10.1186/s12909-022-03206-z)
Supplement: Supplementary file 1 — Additional file 1. [file 12909_2022_3206_MOESM1_ESM.pdf]

Basic Life Support Survey

Investigators:
1. Ssewante Nelson (Makerere University)
2. Wekha Godfrey (Makerere University)
3. Iradukunda Angelique(Makerere University)
4. Musoke Phillip (Makerere University)
5. Jonathan Kajjimu (Mbarara University of Science and Technology)
6. Andrew Marvin Kanyike (Busitema University)
7. Germinah Nabukeera (Kabaale University)
8. Lauben Kamuhangire (King Caesar University)
9. Kisakye Wamala (Kampala International University)
10. Wilson Zziwa (Gulu University)

Introduction
What you need to know about the Survey:
a). You are being asked to participate in a Survey.
b). This consent form explains the survey and your role in the study.
c). Read it carefully and as much time as you need.
d). You can choose not to take part and if you join, you can quit at any time. No penalty for refusal to participate since it's voluntary participation.

Brief Background
Uganda loses more than 3,000 people annually to road traffic accidents. Other Non-Communicable Diseases such as cardiovascular diseases including ischemic heart disease and stroke which predispose people to Sudden Cardiac Arrest are also on a rise. Regardless of this notion, however, knowledge and practice of Basic Life Support among health workers remain to be determined.
With Uganda still lacking a well-established emergence response system, all front-line health workers must be equipped with basic skills right from training curricula hence the need for this assessment.

Institutions involved;
Makerere University (MAK), Gulu University, Kampala International University, Mbarara University of Science and Technology, Soroti University, Kabale University, Uganda Christian University, Busitema University, King Caesar International University, and Islamic University In Uganda.

Who is eligible;
All students from the above universities on the following programs;
Bachelor of Medicine and Bachelor of surgery
Bachelor of Pharmacy
Bachelor of Nursing
Bachelor of Dental Surgery

Benefits
Findings from this study will be shared with the above institutions with recommendations to incorporate Basic life support into their respective curriculum.

In case of some questions about the survey, who do I contact?

You can contact the Principal investigator
Nelson Ssewante: 0706400240/782937220, nelson.ssewante1@gmail.com

You may also contact the Chairperson of Mulago Research Ethics Committee, for any issues concerning the human rights of the study participants.
Dr. Fredrick N. Nakwagala at +256772325869

* Required

1. Do you agree to participate in this survey? *

Mark only one oval.

- Yes
- No

Background Information

Please note that this information is not to identify you but rather to provide a basis of analysis. Choose one option for each question.

2. 1. How old are you? *

Mark only one oval.

- 18-25 years
- 26-35 years
- >35 years

3. 2. What is your biological sex? *

Mark only one oval.

- Male
- Female

4. 3. Which university do you belong to? *

Mark only one oval.

- Makerere University
- Mbarara University of Science and Technology
- Gulu University
- Busitema University
- Soroti University
- Kampala International University
- Islamic University In Uganda
- King Ceasar University
- Uganda Christian University
- Kabale University

5. 4. What is your study course? *

Mark only one oval.

- Bachelor of Medicine and bachelor of Surgery
- Bachelor of Nursing
- Bachelor of Dental Surgery
- Bachelor of Pharmacy

6. 5. What is your year of study? *

Mark only one oval.

- Year 1
- Year 2
- Year 3
- Year 4
- Year 5

7. 6. Have you ever attended any BLS training? *

Mark only one oval.

- Yes
- No

Knowledge Assessment

8. 1. When you find someone unresponsive in the middle of the road, what will be your first response? (Note: You are alone there) *

Mark only one oval.

- Open airway
- Start chest compression
- Ensure safety
- Give two breathings

9. 2. If you confirm somebody is not responding to you even after shaking and shouting at him/her, what will be your immediate action? *

Mark only one oval.

- Start CPR
- Activate EMS
- Put him in recovery position
- Observe

10. 3. What is the location for chest compression? *

Mark only one oval.

- Left side of the chest
- Right side of the chest
- Centre of the chest on lower half of breast bone
- Xiphisternum

11. 4. What is the location for chest compression in infants? *

Mark only one oval.

- One finger breadth below the nipple line
- At the intermammary line
- One finger breadth above the nipple line
- At Xiphisternum

12. 5. How do you give rescue breathing in infants? *

Mark only one oval.

- Mouth-to-mouth with nose pinched
- Mouth-to-mouth and nose
- Mouth-to-nose only
- Mouth-to-mouth without nose pinched

13. 6. What is the depth of compression in adults during CPR? *

Mark only one oval.

- At least 2 inches
- 2½ – 3 inches
- 1 – 1½ inches
- 1½ inch

14. 7. What is the depth of compression in Children during CPR? *

Mark only one oval.

- 2 inches
- 2 - 2½ inches
- 1 - 1½ inches
- ½ – 1 inch

15. 8. At what rate should chest compression in adults and Children be performed during CPR? *

Mark only one oval.

- At least 100 / min
- Approximately 100 / min
- At 80 / min
- At 120 / min

16. 9. The Chain of Survival include all of the following EXCEPT? *

Mark only one oval.

- Early CPR
- Integrated post cardiac arrest care
- Advanced airway placement
- Rapid defibrillation

17. 10. How often should rescuers switch roles when performing 2-rescuer CPR? *

Mark only one oval.

- After each cycle
- After 2 cycles
- After 5 cycle
- After 10 cycles

18. 11. The correct sequence for initiation of BLS is; *

Mark only one oval.

- Assess the victim, give 2 rescue breaths, defibrillate, start CPR
- Assess the victim, activate EMS & get AED, check pulse, start CPR
- Check pulse, give rescue breaths, assess the victim, defibrillate
- Assess the victim, start CPR, give 2 rescues breaths, defibrillate

19. 12. In an emergency situation, where should you attempt to perform a pulse check in an adult? *

Mark only one oval.

- Carotid
- Brachial
- Radial
- Temporal

20. 13. The compression to ventilation ratio for the lone rescuer giving CPR to victims of ANY age is: *

Mark only one oval.

- 15:1
- 15:2
- 30:1
- 30:2

21. 14. The proper steps for operating an AED are: *

Mark only one oval.

- On the AED, attach electrode pads, shock the patient, analyze the rhythm
- On the AED, attach electrode pads, analyze the rhythm, clear the patient, deliver shock
- Attach electrode pads, check pulse, shock patient, analyze rhythm
- Check pulse, attach electrode pads, analyze rhythm, shock patient

22. 15. The Guidelines for CPR recommended BLS sequence of steps are: *

Mark only one oval.

- Chest compressions, Airway, Breathing
- Airway, Breathing, Check Pulse
- Airway, Breathing, Chest Compressions
- Chest compression, Airway placement, Breathing

23. 16. Signs of severe airway obstruction include all of the following EXCEPT? *

Mark only one oval.

- Poor air exchange
- High-pitched noise while inhaling
- Unable to cry
- May wheeze between coughs

24. 17. If you and your friend are having food in a canteen and suddenly your friend starts expressing symptoms of choking but responsive, what will be your first response? *

Mark only one oval.

- Give abdominal thrusts
- Give chest compression
- Confirm foreign body aspiration by talking to him
- Give back blows

25. 18. The critical characteristics of high-quality CPR include which of the following? *

Mark only one oval.

- Starting chest compressions within 10 seconds of recognition of cardiac arrest
- Push hard, push fast
- Minimize interruptions
- All the above

Attitudes

26. 1. Basic Life Support (BLS) training is necessary *

Mark only one oval.

- Strongly agree
- Agree
- Neutral
- Disagree
- Strongly disagree

27. 2. In an emergency situation, I would voluntarily perform BLS *

Mark only one oval.

- Strongly agree
- Agree
- Neutral
- Disagree
- Strongly disagree

28. 3. In the event of cardiac arrest, I would initiate chest compressions even without PPEs. *

Mark only one oval.

- Strongly agree
- Agree
- Neutral
- Disagree
- Strongly disagree

29. 4. BLS training should be a part of your curriculum *

Mark only one oval.

- Strongly agree
- Agree
- Neutral
- Disagree
- Strongly disagree

This content is neither created nor endorsed by Google.

Google Forms
